# Climate change affecting oil palm agronomy, and oil palm cultivation increasing climate change, require amelioration

**DOI:** 10.1002/ece3.3610

**Published:** 2017-11-30

**Authors:** R. Russell M. Paterson, Nelson Lima

**Affiliations:** ^1^ CEB—Centre of Biological Engineering University of Minho Braga Portugal; ^2^ Department of Plant Protection Faculty of Agriculture Universiti Putra Malaysia Selangor D.E Malaysia

**Keywords:** *Elaeis guineensis*, *Ganoderma*, global warming, Indonesia, Malaysia, peat

## Abstract

Palm oil is used in various valued commodities and is a large global industry worth over US$ 50 billion annually. Oil palms (OP) are grown commercially in Indonesia and Malaysia and other countries within Latin America and Africa. The large‐scale land‐use change has high ecological, economic, and social impacts. Tropical countries in particular are affected negatively by climate change (CC) which also has a detrimental impact on OP agronomy, whereas the cultivation of OP increases CC. Amelioration of both is required. The reduced ability to grow OP will reduce CC, which may allow more cultivation tending to increase CC, in a decreasing cycle. OP could be increasingly grown in more suitable regions occurring under CC. Enhancing the soil fauna may compensate for the effect of CC on OP agriculture to some extent. The effect of OP cultivation on CC may be reduced by employing reduced emissions from deforestation and forest degradation plans, for example, by avoiding illegal fire land clearing. Other ameliorating methods are reported herein. More research is required involving good management practices that can offset the increases in CC by OP plantations. Overall, OP‐growing countries should support the Paris convention on reducing CC as the most feasible scheme for reducing CC.

## 1 INTRODUCTION

**Figure 1:**
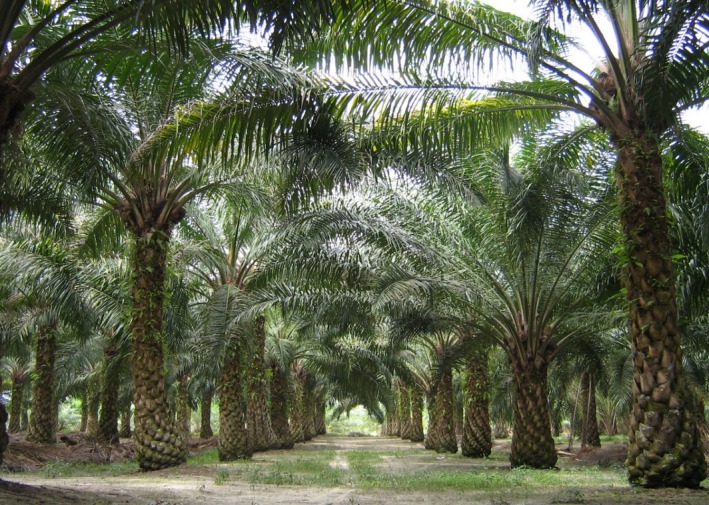
Oil palms within a plantation

Large‐scale oil palm (OP) cultivation (Figure 1) has transformed tropical regions, people's lives, and palm oil companies’ profits. High output, easy establishment, and low costs make the crop very profitable and the most efficient oil crop economically (Dislich et al., 2017). Palm oil use is high and growing rapidly which is driven by economic development in countries such as India and China. Very large oil yields are obtained, and the high adaptability of OP is valuable especially in the climate change (CC) context. Indeed, palm oil is a huge global industry, worth over USD 50 billion annually (Murphy, 2014). Most crude palm oil is used in food and biodiesel production, while palm kernel oil is employed in detergents, cosmetics, plastics, and chemicals. Sustainable OP involves challenges (Rival, 2017), and, for example, development on peatlands appears a myth (Evers, Yule, & Padfield, 2017).

Climate change and OP discussions herein are considered as follows: (1) the effect of CC on OP cultivation, and (2) vice versa. This dual nature is particularly interesting. Most scientific literature supports the occurrence of CC caused largely by human industry. OP plantations have reduced ecosystem functioning generally compared to forests (the predominant ecosystem replaced by OP) (Dislich et al., 2017) and contribute to CC. The burning of peat soils for growing OP, which releases stored C as greenhouse gases (GHG) and causes smog, is a unique problem (WRI, 2015).

Palm oil is incorporated into many commodities and is produced, stored, processed, packaged, transported, and prepared before becoming the desired commodity. These processes release GHG. Nevertheless, only the effect on cultivation of OP is considered in this present review (Paterson, Kumar, Shabani, & Lima, 2017; Paterson, Kumar, Taylor, & Lima, 2015). Indonesia and Malaysia produce ca. 83% of palm oil contributing significantly to their economies. Malaysia's palm oil exports add 45% to the world's edible oil needs (Shanmuganathan, Narayanan, & Mohamed, 2014). The concentration of such a high proportion of palm cultivation in Malaysia/Indonesia is somewhat undesirable as cultivation in other countries combats threats from climate and locally adapted pests and pathogens. Further expansion into West Africa and Latin America creates a more secure production system in the longer term in the opinion of Murphy (2014) and a doubling of palm oil production in the next decades is considered feasible from this expansion. However, these estimations of production do not take into account CC (Paterson et al., 2015, 2017) which undermines these assessments, nor do they consider other negative consequences of OP development such as biodiversity loss (Fitzherbert et al., 2008) and most ecosystem functions (Dislich et al., 2017).

Climate change will have a profoundly negative effect on cultivation of OP, especially by 2100 (Paterson et al., 2015, 2017). On the other hand, some ecological functions of OP plantations compared to forests have potentially irreversible global impacts such as reduction in gas and climate regulation (Dislich et al., 2017). The most serious impacts occur when forest is cleared to establish plantations immediately after removal, and especially on peat soils. The reduced ability to cultivate OP may have benefits in ameliorating CC, as, for example, less deforestation may occur because of the reduced ability to grow OP.

The objectives of this paper were to consider the effects of (1) CC on OP cultivation, and (2) OP agronomy on CC. Procedures to ameliorate these interconnected issues are discussed.

## 2 EFFECT OF OIL PALM CULTIVATION ON CLIMATE CHANGE

The environmental impact of the OP industry primarily concerns the conversion of tropical rainforests into plantations. Most of the area for expansion of the OP industry was supplied through forests where the emissions from conversion exceed the potential carbon fixing of OP (Germer & Sauerborn, 2008; Paterson et al., 2015, 2017). Global production of palm oil was ca. 50 million metric tons per year in 2012 which is more than double that of 2000, of which a considerable amount involved deforestation. The highest carbon emitter countries from forest cover loss for (1) Latin America and the Caribbean, (2) Sub Sarah Africa, and (3) South and South‐East Asia were (1) Brazil, (2) Democratic Republic of the Congo, and (3) Indonesia, respectively, at values of 340, 23, and 105 (Teragrams (Tg) C/year) respectively. Malaysia was third highest at 41 Tg C/year (Harris et al., 2012). Indonesia and Malaysia account for high C emissions from deforestation as they are the first and second highest producers of OP. Substantial palm oil production is also already undertaken in Columbia and Nigeria (Paterson et al., 2017). Emissions from OP cultivation in Indonesia accounted for ca. 2%–9% of all tropical land use from 2000 to 2010 (Carlson & Curran, 2013). Indonesia was the world's seventh‐largest emitter of global warming pollution in 2009, and deforestation accounted for about 30% of these emissions (Union of Concerned Scientists, 2013). Also, plantation expansion in Kalimantan, Indonesia, is projected to contribute 18%–22% of the country's 2020 CO_2_ emissions (Carlson et al., 2012). OP production involving deforestation releases global anthropogenic emissions of 6%–17% CO_2_ (Baccini et al., 2012).

Changing forest to OP plantations gives high reductions in gas and climate regulation function (Dislich et al., 2017). OP plantations produce more GHG and volatile organic compounds (VOC), a precursor to tropospheric ozone. The carbon sequestered by OP does compensate for the GHG emitted from land‐clearing fires and land and plantation establishment. VOC, GHG, and aerosol particles emissions during fire periods result in direct and indirect changes of solar irradiation. OP plantations compared to forest leads to higher air and soil temperature and lower air humidity microclimates (Dislich et al., 2017). Indonesia has substantially expanded OP plantations and smallholder agriculture, reducing drastically the area of primary forest, especially in Sumatra, which has the highest primary rainforest cover loss in the country. Forest cover in Riau and Jambi declined from 93% to 38% between 1977 and 2009 which changed microclimatic conditions because forests regulated the climate. Expansion of OP plantations leads to warming of the land surface and increases in air temperature from CC as observed in Sumatra (Sabajo et al., 2017). OP foliage cover is lower, more open, and simpler than tropical rainforest foliage cover: Clearing land for OP plantations and planting OP results in higher surface temperatures (Ramdani, Moffiet, & Hino, 2014). The warming results from reduced evaporative cooling as the main determinant of regulating the surface temperature. Warming induced by land cover change (LCC) exceeded the global warming effect: understanding this effect may support (1) conservation of existing forest policies, (2) planning and expansion of the OP plantations, and (3) afforestation measures. OP may have adapted to the increased temperature, although the increase in land temperature will exacerbate CC (Sabajo et al., 2017). The increase in LCC, which is seldom considered, is a third factor contributing to CC in addition to deforestation and conversion of peat.

CO_2_ is the primary molecule contributing to the GHG from OP plantations and methane (CH_4_) and nitrous oxide (N_2_O) are modest in comparison, although with greater effect per molecule. Land‐clearing fires lead to large releases of CO_2_ from vegetation and soil, particularly so on peat. Fires can indirectly increase emissions by exposing organic‐rich soil layers to rapid decomposition exacerbated by ash increasing peat decomposition. Large amounts of CO_2_ are released during drainage of peat soil to establish plantations by oxidization and decomposition: dissolved organic matter is flushed out of peat soils when they are drained, which then decomposes and releases additional CO_2_ (Dislich et al., 2017).

Oil palm plantations assimilate CO_2_ from the atmosphere acting as a carbon sink, as does any vegetation. Interestingly, OP plantations assimilate more CO_2_ and produce more biomass than forests due to very high fruit production, often used erroneously as an argument in favor of OP. This higher rate of C uptake does not compensate for that released when forests are cleared, as forests have more aboveground and belowground biomass than OP plantations unless very long timescales are considered. The timescales are hundreds of years (Kotowska, Leuschner, & Triadiati, 2015), well beyond the maximum time frame of ca. 80 years considered in Paterson et al. (2015, 2017) in terms of the effect of CC on suitable climate for OP growth for example. Furthermore, OP plantations release more N_2_O into the atmosphere than forests, mainly from fertilizer use: Fires add black carbon, which increase global warming. OP plantations have a direct effect on local microclimates by having lower, less dense canopies and a lower leaf area index than forests (Dislich et al., 2017). Peatland deforestation for OP cultivation in West Kalimantan, Indonesia, increases GHG emissions greatly (Barcelos et al., 2015; Carlson et al., 2012). However, OP plantations managed in a manner harmless to the environment may be sustainable production systems (Sheil et al., 2009). Overall, the biological and managerial tools to surmount many challenges exist but need better support (Murphy, 2014).

## 3 EFFECTS OF CLIMATE CHANGE ON PALM OIL CULTIVATION

There is an increasing awareness of the negative effect of CC on the OP industry (MPOC, 2013; Paterson et al., 2015, 2017). CC will (1) reduce overall the current cultivated areas, (2) extend plantations to new areas, assuming issues such as biodiversity loss are overcome, and (3) challenge the capacity for adaptation by growers. The conditions of OP cultivation by abiotic (i.e., rainfall, temperatures, carbon dioxide, and soil salinity) and biotic (i.e., diseases, pests, pollinators, and associated crops) stresses will be affected detrimentally in most cases (Rival, 2017). Tropical plants are often at the limits of growth, where small changes in climate can affect survival. In general, more crops and greater yields are projected to occur in regions that are cool (e.g., subtropical), while fewer crops and yields are projected to occur in regions that are hot (e.g., tropical) (Paterson et al., 2015).

Palm oil production has already declined because of the direct and indirect uncertainties of CC. Zainal, Shamsudin, Mohamed, and Adam (2012) predicted that palm oil revenue would reduce by 341.29, 127.43, and 51.80 MYR/ha for Peninsula Malaysia, Sabah, and Sarawak, respectively, by 2029. Even greater losses are predicted by 2099 for Sabah and Sarawak (294.20 and 105.62 MYR/ha, respectively) although Peninsular Malaysia was similar. Increase in temperature and rainfall resulted in 41, 49, and 38 MYR/ha decrease for Peninsular Malaysia, Sabah, and Sarawak. These figures are without adaption and mitigation strategies being taken (Zainal et al., 2012) and are to some extent confirmed by decreases in suitable climate for OP growth during similar periods (Paterson et al., 2015, 2017).

Understanding the CC effects on OP (Paterson et al., 2015, 2017) is vital for developing novel cultivation practices and assuring world food security in the palm oil sector. CC effects on OP phenology and fruit production have profound implications at local and international levels. Shanmuganathan et al. (2014) examined the recent CC effects on OP yield between 2007 and 2011. El Niño and La Niña climate events on local climate and OP production in Tumaco, Colombia, established that they had lagged and conflicting impacts on yields. El Niño was favorable, showing a maximum correlation with production 2.6 years after the event. Meanwhile, La Niña caused severe droughts, with the highest reduced yield in 2002. OP are susceptible to drought (Dislich et al., 2017).

High temperatures and heavy rains were favorable to palm oil production in the western coast of Sabah, Malaysia, with a lag period of 3 and 4 months, respectively. Flooding and severe drought were unfavorable in some cases. The higher precipitation/floods of the La Niña decreased the production and quality of crude palm oil (CPO) attributed to affecting the fruit ripening stage and reflected in the yield in subsequent months. CC variability and its effects on OP yield in East and West Malaysia revealed correlations between climate variations, OP tree phenology, and yield. Average monthly temperature 8 months prior to harvest of ≥27.83°C led to low yield across Malaysia (Shanmuganathan et al., 2014). Furthermore, OP yields are projected to decrease by 30% should temperature increase 2°C above optimum and rainfall decreases by 10% in Malaysia. Reduction in CPO production caused by CC in southern Malaysia was 26.3% and drought in SE Asia caused declines of 10%–30% in palm oil production. A temperature variation of 0.6–1.4°C and ±15% rainfall variation led to a positive change in earnings for PO of up to $2,453 per year, while earnings were reduced to $1,181 per year with ±32% rainfall fluctuation and moderate temperature fluctuation. The countries which cultivate OP will face increasing uncertainty in the future (Paterson et al., 2015, 2017).

Brazil, the Democratic Republic of Congo, Indonesia, Peru, and Columbia have an estimated 2.00, 0.78, 0.61, 0.46, and 0.42 million hectares, respectively, of forest identified as suitable for OP growth. However, these countries will experience large decreases in climate suitability (Paterson et al., 2017). OP plantations are limited to low elevation areas and are in direct conflict with tropical lowland forests, including those found within riverine floodplains subject to periodic flooding by rivers or streams. Consequently, unsuitable areas are principally linked to seasonal and/or tidal inundation events. Nevertheless, simplistic biophysical criteria are often used by governments and agencies for agricultural zoning for OP that includes slope, elevation, and soil types within suitable climatic zones which may fail to capture regionalized constraints. In 2011, 1.43 million hectares (19.3%) of Sabah's terrestrial extent was under OP which could increase to 2.1 million hectares by 2025 (Abram et al., 2014). Paterson et al. (2015, 2017) indicate that the current highly suitable climate of Sabah for OP will not decrease until 2100 and so this does not contradict the just‐mentioned prediction for 2025. OP expansion will likely continue to target the eastern State floodplains areas that have very high yield potential. Much of the unproductive OP is related to flooding which is likely to increase with CC (Abram et al., 2014).

**Figure 2:**
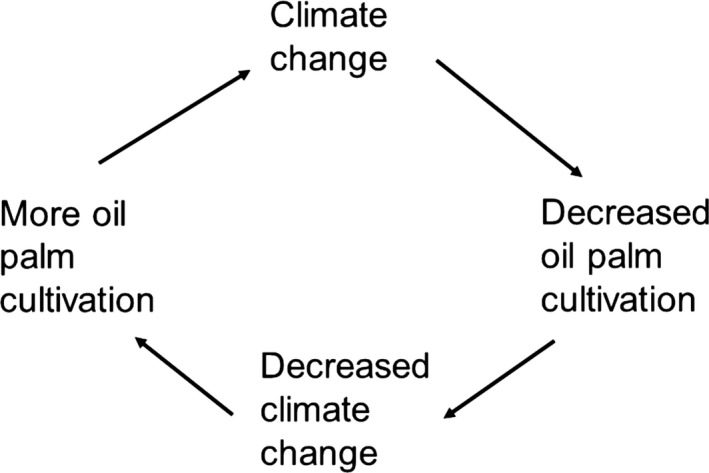
Cyclic nature of the effect of climate change (CC) on oil palm (OP) cultivation. Overall, the tendency will be for progressively reduced levels of OP cultivation. The contribution to world CC from deforestation through OP agronomy is only one factor. Reduced OP cultivation may not have a very large effect of reducing CC per se, but could be significant

Procedures involved in the cultivation of OP increase CC which, in turn, will affect negatively growth of OP, which will reduce CC, etc. in a cyclic process (Figure 2) but tending toward reduced OP growth. The reduction in CC from a decrease in growing OP may not be large.

Finally, Corley and Tinker (2016) state that OP suffering more disease from CC, as mentioned in Paterson, Sariah, and Lima (2013), appears unjustified. However, the premise that stress conditions (Paterson et al., 2015, 2017) caused by CC is likely to increase OP disease is justified in the current authors’ opinion. Furthermore, Rival (2017) implies that, *inter alia*, CC will increase diseases and pests of OP, hence corroborating the premise. Paterson et al. (2013) provide extensive information on crop disease decreases and increases linked to CC, although there is little published on the effect on OP disease of CC per se: This effect on disease will become more apparent in the future.

## 4 REDUCING CLIMATE CHANGE BY ADAPTING OIL PALM CULTIVATION

Plantation management measures can prevent or reduce losses of some ecosystem functions which will reduce CC. These include (1) avoiding illegal land clearing by fire, (2) avoiding draining of peat, and (3) using cover crops, mulch, and compost (Dislich et al., 2017). Reducing GHG by limiting OP expansion to areas with moderate or low carbon stocks is most effective. This involves ceasing development of plantations on peatland and enforcing the moratorium on new concessions in primary forests. Governmental policy in Indonesia prohibits the clearing of land by burning, but this is not always enforced, and such enforcement would be a positive step. In addition, rehabilitation and restoration of converted peatlands are an option. Establishing new OP plantations only on degraded or existing agricultural land is highly desirable, although which land is acceptable for OP is debatable. Limiting flooding may prevent increased CH_4_ emissions on mineral soils.

Oil palm is scrutinized for its environmental effects, but sustainable cultivation may be possible (Samedani et al., 2015). Reducing unnecessary expansion of plantations and ensuring existing ones are managed optimally are crucial. The large CO_2_ fluxes from tropical peatlands play an important role in global CC and promoting policies and strategies to manage them more sustainably is important. Mechanisms such as (1) reduced emissions from deforestation and forest degradation, plus conservation, sustainable management of forests, and enhancement of forest carbon stocks (REDD+), (2) national greenhouse gas accounting, and (3) accurate emission factors for C dynamics are essential (Comeau et al., 2016). Growth of the OP industry may occur on land presently covered by lowland forest, degraded grassland, and agricultural land currently under alternative uses, to avoid conversion of forests and peatland (Germer & Sauerborn, 2008). Plantations are replacing grassland or scrub where the average C content of the plantation will exceed that of the previous vegetation and so becoming a greater C sink.

### 4.1 Disease control

Controlling disease may assist in decreasing the unwanted expansion of plantations as yields will be increased from reduced disease in current plantations, such as described for *Ganoderma* rots of OP (Mohd As'wad, Sariah, Paterson, Zainal Abidin, & Lima, 2011) (Muniroh, Sariah, Zainal Abidin, Lima, & Paterson, 2014). The current awareness of environmental issues makes optimizing current plantations by reducing disease imperative in any case. However, increasing the profitability of existing plantations may provide motivation to owners for expansion: This concept may require greater discussion but is beyond the scope of the current review.

### 4.2 Fertilizer control

Reducing nitrogen fertilizer decreases nitrogen‐based emissions (Dislich et al., 2017). OP plantations release large quantities of nitrous oxide (N_2_O) into the atmosphere linked to nitrogen (N) fertilizer use. More work is required on comparing effects of soil (see below) and N fertilizer on N_2_O and CO_2_ emissions. Sakata et al. (2015) demonstrated that N_2_O and CO_2_ fluxes in OP plantations were significantly affected by the type of soil, but not always by fertilizer treatments: Simunjan sandy soil was lowest for N_2_O emissions, and Tatau peat soil was the highest. The data on N application and respiration rate are variable and require determinations for particular biomes (Zhong, Yan, & Shangguan, 2016). Increased flux of CO_2_ after N fertilizer application was observed occasionally and confirmed rapid emission enhancement in a matter of days following fertilizer application in tropical peatlands, hence potentially increasing CC. More work is required using different systems (Comeau et al., 2016).

### 4.3 Role of different soils

An option for OP planting, without threatening tropical rain forests, is the rehabilitation of anthropogenic grassland, created by human clearance of natural forest eons ago. There exist vast areas of anthropogenic grassland in Indonesia where much of the spread of OP plantations will take place. “Flexibility mechanisms” could act as an incentive for grassland rehabilitation. The biomass of tropical lowland forests, the forest type most frequently converted to OP growing, is usually higher than that of upland forest, reflecting the high soil fertility and favorable rainfall in areas suitable for OP production. C fixation in plantation biomass and soil organic matter results in the net removal of ca. 135 Mg CO_2_ per hectare from the atmosphere when tropical grassland is rehabilitated by OP plantations. Conversely, emission from forest conversion exceeds the potential carbon fixation of OP plantings. Grassland rehabilitation may (1) preserve natural forest, (2) avoid emissions, and (3) generate additional revenue if the sequestered C becomes tradable (Germer & Sauerborn, 2008).

### 4.4 Reduced emissions from deforestation and forest degradation

Considerable funding has been obtained for the REDD+ (http://redd.unfccc.int/) scheme. Nevertheless, meaningful emission reduction or revenues from C credits have not been achieved in Indonesia at least. Earlier initiatives have shown promising results, albeit slowly, in the number of companies moving toward certification of forest management (Dermawan, Sinaga, Williams, Standing, & Dupuy, 2015). REDD+ proposals include growing OP on reclaimed soil and replacing the use of fertilizer with other methods. Overall, the REDD+ scheme is still being developed where the apparently worthwhile proposals need carrying out in a verifiable manner.

Global analysis suggests in a few cases that OP may encourage forest reversion and lower global emissions, mainly because OP plantations store more carbon than alternative agricultural land uses. However, this is valid only where degraded lands, such as grassland in Indonesia and cattle pastures in the Amazon, are used for OP cultivation; a solution embraced by environmentalist and policymakers (WRI, 2015). OP is even more acceptable if policymakers: (1) stop deforestation, (2) introduce peatland restoration policies, (c) support smallholder farms, and (d) involve local communities in palm oil business. Thus, OP cultivation alleviates poverty and could transform livelihood of millions of people with suitable governmental policies (Barcelos et al., 2015).

## 5 AMELIORATING THE EFFECT OF CLIMATE CHANGE ON OIL PALM PRODUCTION

Strategies are required to minimize the adverse effects of CC on OP cultivation. These practices may also decrease CC from less deforestation if the yields of existing OP are optimized to cope with CC.

### 5.1 Develop oil palm in novel regions

The concentration of ca. 85% of OP cultivation in Malaysia/Indonesia is detrimental to combating CC. More dispersed cultivation outside these countries could ameliorate threats from CC as a wider range of climates would be encountered, some of which may be more suitable for OP. The expansion into West Africa and South/Central America underway was intended to create a more secure production system in the longer term, coupled with the reduced available land in Malaysia and Indonesia (Murphy, 2014). However, Paterson et al. (2015, 2017) demonstrated that Latin America and Africa may be even more affected by CC in terms of suitable climate for growing OP than SE Asia, meaning that this expansion is unlikely. The increase in biodiversity loss and decreases in ecological functions previously mentioned would also mitigate against expansion into novel areas.

### 5.2 Growing oil palms in novel suitable regions created by climate change

Cultivation at higher altitudes and/or lower and higher latitudes may be possible beyond the lowland tropics as CC progresses (Paterson et al., 2017). Paterson et al. (2015) predicted an increase in highly suitable climate (HSC) for growing OP by 2030 in Indonesia and Malaysia largely in mountainous regions of Sumatra, Sarawak, Borneo, and Sulawesi. These areas had increasingly HSC by 2070 and 2100 being almost the only more suitable regions amidst the general decrease. There may other factors which do not permit OP growth, for example, lack of suitable soil, which require further investigation. The other factors mitigating against employing this novel cultivation area may include decreased biodiversity and ecological function (see above).

Table 1 demonstrates the averages of four data sets (Paterson et al., 2015) illustrating trends in the change of suitable climate more clearly. The areas for HSC are in factors of 10^6^ km^2^, whereas those for the other area types are, at most, at a factor of 10^5^. There is a slight increase, and medium, and large decreases in HSC in 2030, 2070, and 2100, respectively. These changes are reflected in corresponding changes in unsuitable, marginal, and suitable climates. The most significant figure is the large decrease in HSC by 2100 of 7.9 × 10^5^ km^2^ (56%). Concomitant increases of 4.70 × 10^5^ km^2^ (7778%) and 5.25 × 10^5^ km^2^ (6738%) in regions with marginal and suitable climate, respectively, were determined and unsuitable climate regions decreased by 2.03 × 10^5^ km^2^ (39%). The increase in marginal and suitable regions was derived from the HSC regions largely and the unsuitable regions to a lesser degree. Hence, there may be novel areas for OP development even under CC, although in general, the climate suitability *per se* will be reduced, especially from the dominant HSC currently experienced. The marginal climates may not be suitable for OP resulting in low yields and disease (see also below).

**Table 1:** Changes in areas with suitable and unsuitable climate in Malaysia and Indonesia combined. N.B. only the highly suitable category is at the high levels of 10^6^ which is the predominant situation in the countries currently

Scenario	Area (km^2^)			
	Unsuitable × 10^5^	Marginal	Suitable	Highly suitable × 10^6^
Current	3.32	6.12 × 10^3^	7.91 × 10^3^	1.79
2030	2.27	1.01 × 10^4^	3.41 × 10^4^	1.87
2070	1.39	5.67 × 10^4^	2.74 × 10^5^	1.67
2100	1.29	4.76 × 10^5^	5.33 × 10^5^	1.00

Paterson et al. (2017) considered Malaysia and Indonesia separately and a similar pattern was observed with a dramatic decrease in HSC by 2100, from marginal and suitable climate areas. HSC decreased by 100% in Indonesia, whereas unsuitable, marginal, and suitable increased by 36%, 98%, and 96%, respectively, by 2100. In Malaysia, HSC decreased by 271%, whereas marginal and suitable increased each by 100%. Unsuitable climate decreased by 70%. Overall, there is a general decrease in HSC to less suitable climate which will have a detrimental effect on yields, including a probable increase in diseases from additional climate stress (Paterson et al., 2013).

Nevertheless, there is scope for growing OP in the reduced or novel HSC regions and the newly suitable climate regions despite CC, although the marginal climate areas are unlikely to support OP. A caveat being potential biodiversity and ecological function loss if novel areas are converted from, for example, forest. A premium will be placed on being able to grow OP in suboptimal conditions, for example, by breeding for new varieties (Rival, 2017) and/or ensuring that conditions are as suitable as possible for growth of the palms as discussed herein. Detailed descriptions of where the climate becomes more apt for OP are provided in Paterson et al. (2017), which are often in countries which do not grow large amounts of OP currently. In the medium and long term, a considerable geographical extension of OP cultivation in a broad zone across the tropics of Africa, Asia, and the Americas (Murphy, 2014) may not be possible, although there may be small increases in some currently suboptimal regions, when considering ability to grow OP only.

Paraguay will have high increases in HSC and suitable climate by 2050 which is maintained until 2100, despite not being an important OP grower currently, whereas Madagascar will have increases in HSC during these periods (Paterson et al., 2017). Argentina, Southern Brazil, South Africa, Manamer, Bangladesh, and southern China had increases in HSC by 2100, but which was not observed in 2050. Hence, it may be possible to expand OP cultivation in these countries and regions, although biodiversity and ecological function loss should play major parts in deciding the feasibility of using these novel areas. Indonesia, Malaysia, and Columbia have increases in merely suitable climate and marginal climate despite decreases in HSC, and so may be able to continue with cultivating OP, although with greater climate stress. Thailand, Brazil, and Nigeria are considered to change directly to unsuitable climate from HSC and so have less scope for adaption. Brazil has not fully developed its current potential to produce palm oil and so may not be as affected by the CC problem, although expansion of the crop appears limited by CC as discussed herein.

### 5.3 Landscape management

Landscape management can have a positive influence on soil biodiversity and ecosystem functioning, such as maintaining riparian reserves and integration of cattle into OP plantations (Abram et al., 2014) (Tao, Slade, Willis, Caliman, & Snaddon, 2016). These will have benefits for combating CC.

#### 5.3.1 Cover crops to reduce climate effects

Negative microclimatic effects associated with clear‐cutting senescent plantations can be mitigated by sequential replanting that leaves a range of palm ages and maintains canopy cover (Dislich et al., 2017). The sustainability of OP production will depend in part on using cover crops, especially under suboptimal conditions. Leguminous cover crops are grown to (1) coexist with OP following jungle clearing and planting/replanting, (2) provide complete cover to an otherwise bare soil, and (3) protect from erosion. Leguminous cover crops also perform multiple functions such as reducing soil water evaporation, reducing runoff losses, improving/maintaining soil fertility, and recycling of nutrients (Samedani et al., 2015). Leguminous crops benefit subsequent crops by (1) the addition of N, (2) disease and weed control, and (3) improved soil water‐holding capacity. These promise reduced environmental pollution and improved crop yields. Legumes may reduce C and N losses from OP systems and increase soil C sequestration. Some examples for OP are as follows: Pigeon pea, Calopo, butterfly pea, white tephrosia, and Brazilian stylo some of which are already in use in SE Asia (Samedani et al., 2015).

#### 5.3.2 Soil management

The effects of soil management practices including (1) empty fruit bunch (EFB) application, (2) palm frond application and chemical fertilization improving soil fauna (worms, beetles, and ants) feeding activity, and (3) better soil chemical properties show considerable promise. EFB greatly enhanced soil fauna feeding activity and is associated with increased concentrations of base cations and soil moisture. The elevated biological activity has high potential to assist ecosystem functions such as litter decomposition, nutrient cycling, organic carbon stabilization, and ultimately OP productivity. These are factors that would also be useful in ameliorating CC and enhancing OP growth under suboptimal conditions. The application of crop residue in OP ecosystems may have a role in (1) enhancing soil resilience to CC effects, such as drought and flooding, and (2) ameliorating disturbances associated with second and third replanting cycles in South‐East Asia (Tao et al., 2016). However, the possibility of EFB contributing an inoculum for disease such as *Ganoderma* stem rot requires consideration (Kalidas & Sravanthi, 2014; Paterson, Holderness, Kelley, Miller, & O'Grady, 2000).

The use and presence of earthworms may increase the effectiveness of growing OP, as they can contribute to soil turnover, structure formation and serve as a fertility enhancer (Sabrina, Hanafi, Azwady, & Mahmud, 2009). They have been recommended to improve crop health and suppress diseases in general (Elmer, Street, Box, & Haven, 2012). This biological factor should not be overlooked as a means to combat the effects of CC.

### 5.4 Developing oil palm varieties resistant to climate change

Breeding OP for CC requires multidisciplinary and collaborative research (Rival, 2017). The identification of OP genetic variation in response to stress is required, implying the exploration of resources provided by natural variation, germplasm collections, selected genitors from breeding programs, and material of interest collected from smallholders. Hence, one can immediately anticipate how complicated, lengthy, and expensive this process may be.

Paterson, Moen, and Lima (2009) suggested developing OP with high lignin content as a way of combating disease by the white rot fungus *Ganoderma*, thereby making OP more resistant to CC, as disease is considered to increase with CC (Paterson et al., 2013; and above). However, Murphy (2014) mentions a possible way of increasing palm oil yield is to channel more C toward lipid biosynthesis, and less toward other “less valuable” end products such as lignin. The majority of C assimilated via photosynthesis produces a lignified trunk that has relatively little economic value (Murphy, 2014). This is deceptive as lignin protects from disease as mentioned, especially when it is the white rot fungus *Ganoderma* (Paterson, 2007). Selecting for complete resistance, rather than tolerance to diseases, leads to high selection pressures for new variants of the pest/pathogen that can overcome the resistance in the crop. Sequencing of the OP and disease genomes may assist (1) greatly in the identification of genes related to virulence and (2) breeders to develop more tolerant varieties of OP, and/or (3) developing lower virulence strains of *Ganoderma* to outcompete high‐virulence strains (Murphy, 2014). Zainal et al. (2012) recommend the development of OP varieties tolerant to high temperatures and which utilize low amounts of water. Also, understanding how CC affects (1) chemical and physical processes in soils, (2) nutrient availability, and (3) changed availability of nutrients will influence OP breeding programs (Rival, 2017).

Nevertheless, it will be difficult to develop OP resistant to CC partly because it is not known precisely how climate will change. Paterson et al. (2015, 2017) provide information on the types of stress involved. New regions will become increasingly suitable for OP cultivation with CC although with a risk that novel disease may threaten the crop (Rival, 2017). This is supportive to the hypothesis that CC will cause more disease. The Parasites lost phenomena should be considered where crops planted in new regions may have fewer pests and diseases (Paterson et al., 2013).

High fertilizer use causes increased emissions of GHG from fertilizer manufacturing, transportation, and application, and so improvements will be required in the OP nutrients uptake efficiency by breeding for suitable root systems. Prolonged root uptake and better remobilization of nutrients are targets for breeding, provided there is sufficient plasticity of these characteristics in the OP (Rival, 2017).

## 6 AMELIORATING CLIMATE CHANGE EFFECTS ON OP PRODUCTION AND DECREASING CLIMATE CHANGE FROM OP CULTIVATION

A dual effect can be obtained of reducing (1) the effect of CC on OP growth, and (2) CC caused by OP cultivation in the case of some procedures as follows:‐

### 6.1 Arbuscular mycorrhizal fungi

Optimizing the rhizosphere by the use of arbuscular mycorrhizal fungi (AMF) will also assist in reducing CC with generalized benefits to OP growth, by reducing the need for fertilizer for example (Sakata et al., 2015). In general, arbuscular mycorrhizal (AM) symbioses have beneficial effects on water transport to assist in overcoming drought conditions (Augé, Toler, & Saxton, 2015), of relevance particularly to ameliorating the effect of CC. However, few published reports on the interaction of AMF and OP are available. The inoculation of OP seedlings resulted in a threefold growth enhancement compared to noninoculated plants after 570 days in natural soil substrate with no fertilizer addition. The inoculation of OP seedlings with AMF increased plant growth and nutrient uptake of OP and in particular P uptake was enhanced by 37%–44%. Application of AM, as single (*Glomus* sp*.)* or mixed species (*Acaulospora* sp., *Gigaspora* sp., *Glomus* sp., *Scutellospora* sp.), demonstrated better growth performance compared to that of chemical fertilizers (Naher, Othman, & Panhwar, 2013). Reducing fertilizer production and use will cause decreased emissions that lead to CC, and the use of AM could ameliorate the effects of CC on OP.

### 6.2 Char to sequester CO_2_

“Slash‐and‐char” as an alternative to “slash‐and‐burn” of forests cleared for OP may be beneficial and feasible. Slash‐and‐char effectively produces charcoal as a method to sequester CO_2_ normally employed for forest residues. This could be used more extensively to improve agriculture in the humid tropics, enhancing local livelihoods and food security, while sequestering C to mitigate CC (Sheil et al., 2009, 2012). Significant waste is produced from crop residues such as (1) forest residues, (2) mill residues, (3) field crop residues, and (4) urban wastes in many agricultural and forestry production systems. Many of these can be used to produce biochar and applied to agricultural soil. Up to 12% of the total anthropogenic C emissions by land‐use change can be offset annually in soil if slash‐and‐burn is replaced by slash‐and‐char (Lehmann, Gaunt, & Rondon, 2006). Biochar soil management systems can deliver tradable C emissions reduction as the C sequestered is accountable and verifiable.

New Terra Preta‐type sites could be the basis for sustainable OP including under CC, in, for example, Indonesia. This could combat desertification, sequester atmospheric CO_2_ in the long term, and help to maintain biodiversity in tropical rainforests. Large‐scale generation and utilization of Terra Preta soils would decrease the pressure on primary forests that are being extensively cleared for OP growth, but with only limited fertility and sustainability and, hence, providing a limited time for cropping. This would mitigate land degradation and CC. However, the infertility of most tropical soils (and associated low population density) is what could have prevented clearance of tropical forests for agriculture in the first place. Increased fertility may increase the populations supported by shifting cultivation, thereby maintaining and increasing pressure on forests (Glaser, 2007). Interestingly, forested *Terra Preta* locations support above average densities of palms, although not necessarily OP, in the Amazon (Sheil et al., 2012).

Biochar can be produced from large industrial facilities to the individual farm and domestic level (Woolf, Amonette, Alayne Street‐Perrott, Lehmann, & Joseph, 2010). The fraction of the maximum sustainable technical potential that is actually realized will depend on socioeconomic factors, including the extent of government incentives and the emphasis placed on energy production relative to CC mitigation. Overall, the extent to which biochar can be employed remains debatable.

### 6.3 Tillage

Reduced tillage is another possibility for affecting CC, where reducing tillage with AMF provides the optimal conditions for OP. Low tillage in combination with AMF assists nutrient uptake, water relations, and protecting against pathogens and toxic stress (Naher, Othman, & Panhwar, 2013), hence potentially ameliorating the effect of CC on OP growth. Also, low tillage will decrease the emission of GHG from OP plantations (Sakata et al., 2015), hence decreasing CC.

## 7 GENERAL DISCUSSION

Biodiversity loss by developing novel plantations will inhibit further expansion in Latin America and Africa. OP expansion in novel biodiversity‐rich regions such West Africa and Latin America that lead to further major deforestation in those regions will simply exacerbate the environmental problem experienced in SE Asia and requires avoiding. Government action in particular is needed to ensure environmental issue receives as much weight as economic. CC appears inevitable and even more government action will be required to reduce these alterations. CC will have a profoundly negative effect on biodiversity and ecosystem function as is generally well known. However, this present review does not concern the effects of CC generally but is specific to OP.

The amelioration procedures mentioned herein will require to be proven by further experimentation in some cases. However, it is unknown whether OP companies and smallholders will employ them. Further work by NGOs, accountants, sociologist, and government will be required if they are to be implemented. This paper is intended to contribute to the discussion, and the procedures may incur additional cost to the overall operation. The recommended procedures can be incorporated into existing certification schemes. Finally, large‐scale oil palm monoculture plantations must be under control across the tropics.

## 8 CONCLUSIONS

Current results indicate a reduction in climatic suitability for OP production worldwide which are gradual by 2030, and more pronounced by 2100. These imply that palm oil production will be severely affected by CC, with obvious implications for the economies of Indonesia and Malaysia and for the international manufacture of palm oil products. The growth of OP might become optimal in currently subtropical regions as a consequence of the general movement of crops to the Poles, although biodiversity and ecological function loss require careful consideration in these novel regions. There is a general consensus that as CC progresses the climate suitability for growing crops will move toward the Poles. For example, as the tropics becomes too hot for the growth of crops, suitable climate will progress toward the subtropics further north and south (Paterson et al., 2013, 2015, 2017). However, mitigation is possible as indicated in the current review. Ultimately, the optimal overall strategy to reduce the effect of CC on OP growth is to reduce CC in general and a way forward with considerable hope are the measures in the Paris treaty (http://www.un.org/sustainabledevelopment/climatechange/). This represents an agreement to keep CC controlled which requires cooperation internationally.

## CONFLICT OF INTEREST

None declared.

## AUTHOR CONTRIBUTIONS

RRMP conceived the manuscript, wrote the manuscript, and corresponded with reviewers to produce the manuscript. NL facilitated the production of the paper including providing advice and editorial input.
